# Supplementary figures and images for: 3D Sugar Printing of Networks Mimicking the Vasculature
Source: Micromachines (Basel). 2019 Dec 30;11(1):43. doi: 10.3390/mi11010043 (PMC7019326; doi:10.3390/mi11010043)

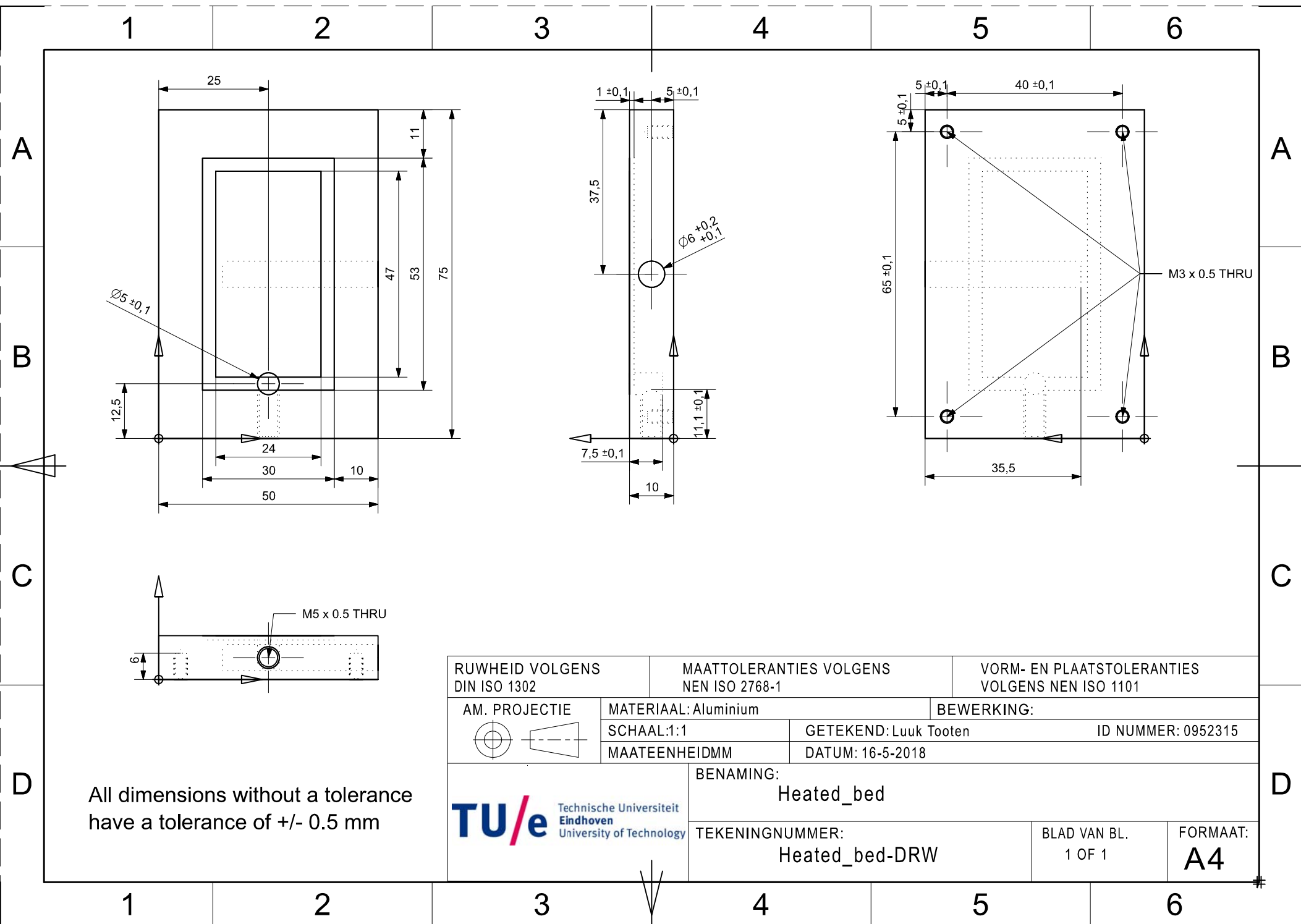

Supplement: Supplementary file 1 [file micromachines-11-00043-s001.zip › Printbed.pdf]

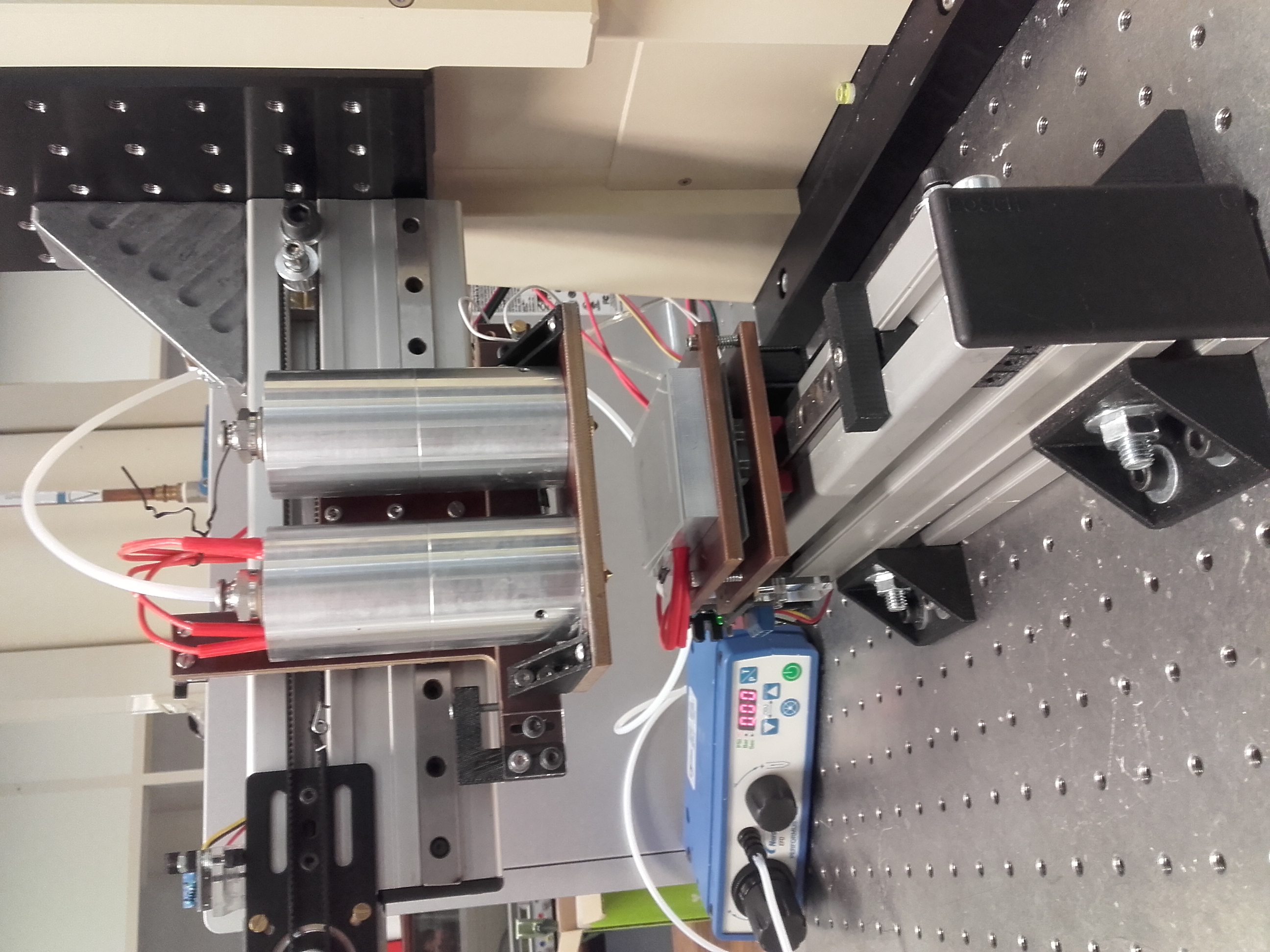

Supplement: Supplementary file 1 [file micromachines-11-00043-s001.zip › Images 3D printer/20180829_101404.jpg]

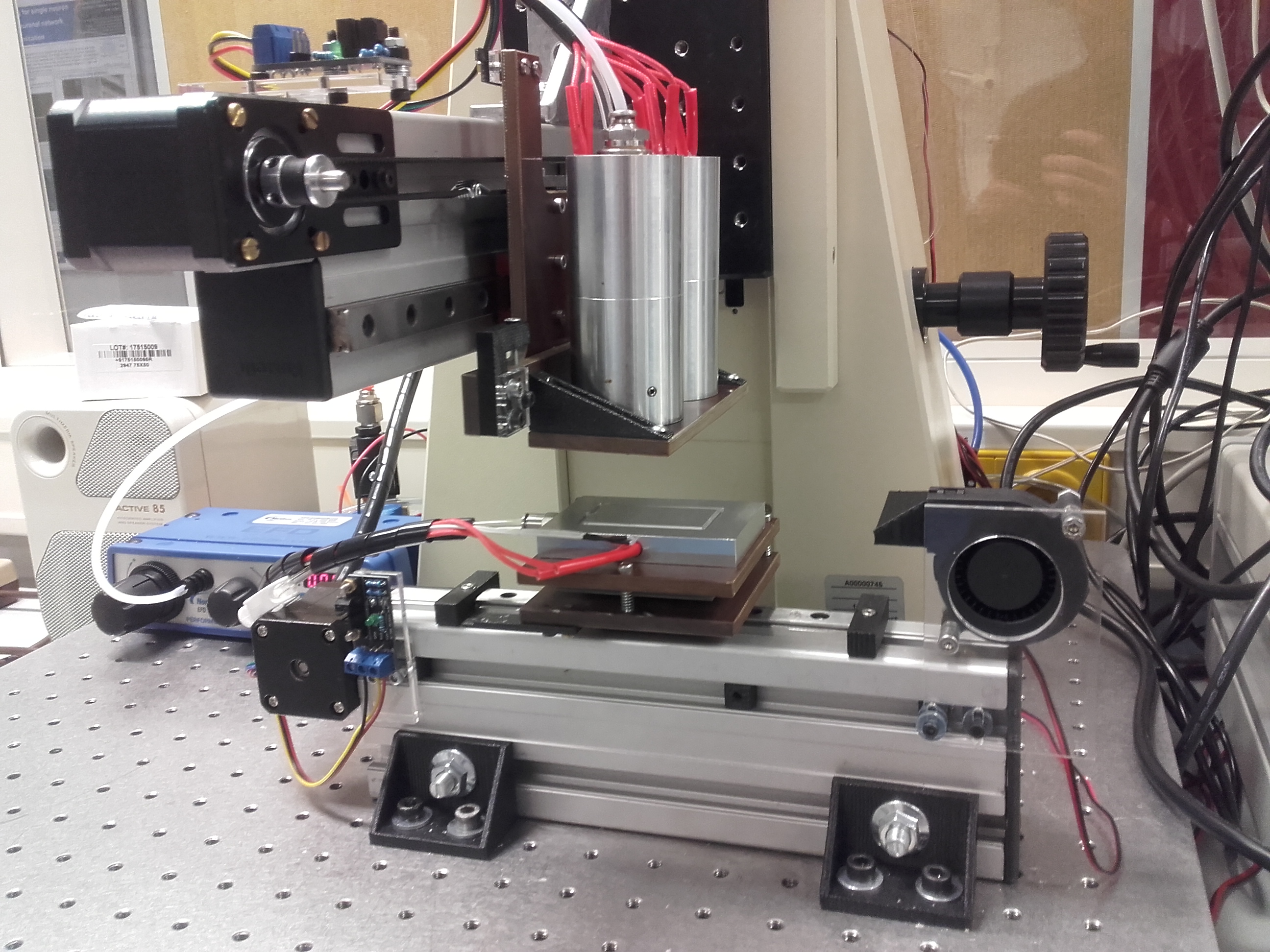

Supplement: Supplementary file 1 [file micromachines-11-00043-s001.zip › Images 3D printer/20181008_140222.jpg]

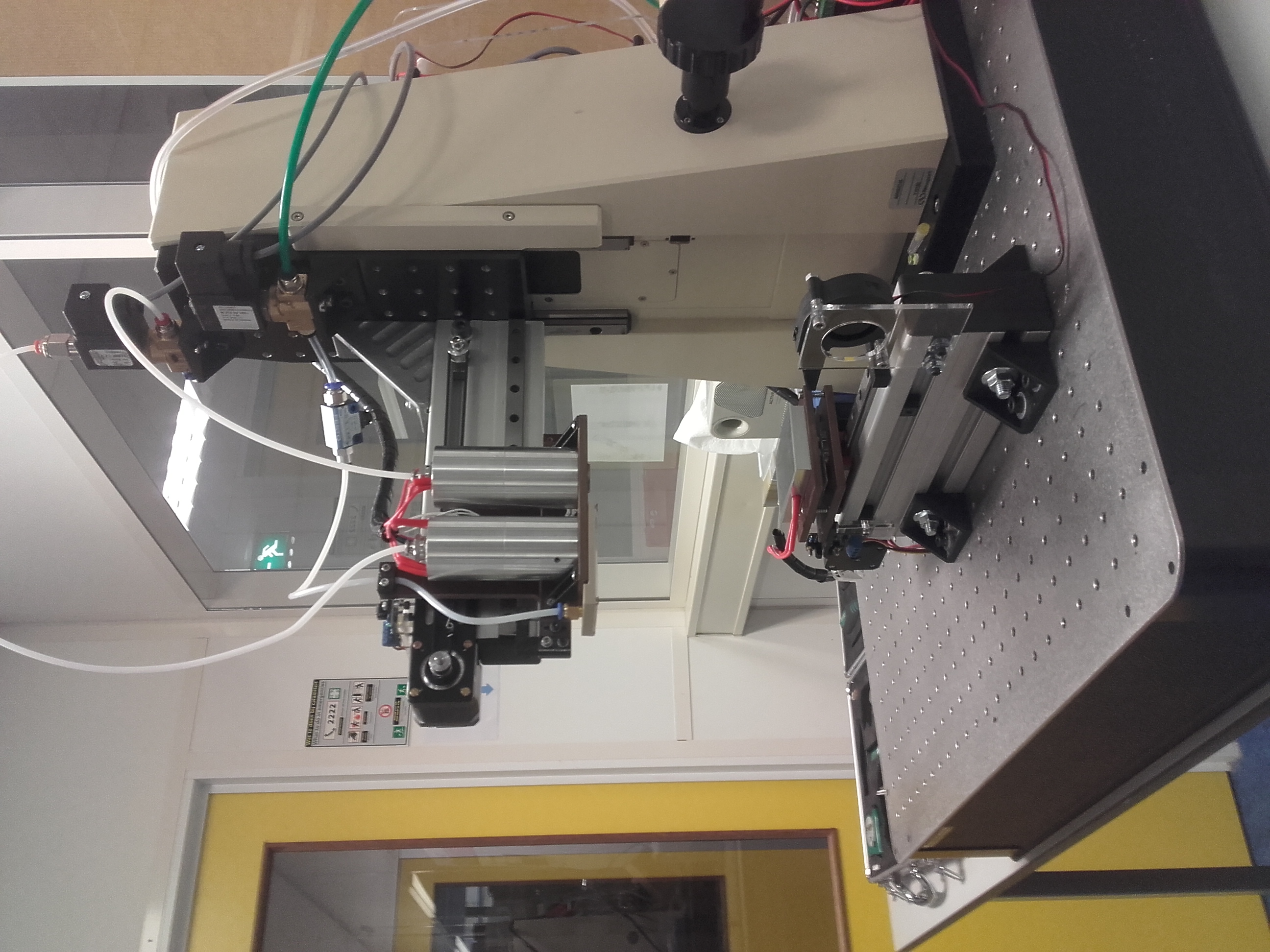

Supplement: Supplementary file 1 [file micromachines-11-00043-s001.zip › Images 3D printer/20190213_165432.jpg]
