# Supplementary material for: 3D Sugar Printing of Networks Mimicking the Vasculature
Source: Micromachines (Basel). 2019 Dec 30;11(1):43. doi: 10.3390/mi11010043 (PMC7019326; doi:10.3390/mi11010043)
Supplement: Supplementary file 1 [file micromachines-11-00043-s001.zip › e3D nozzle dimensions.pdf]

Copyright (C) E3D-Online Ltd (e3d-online.com)

These drawings are free hardware: you can redistribute it and/or modify it under the terms of the GNU General Public License as published by the Free Software Foundation, either version 3 of the License, or (at your option) any later version.

THIRD ANGLE PROJECTION
ALL DIMENSIONS IN MM
MATERIAL: AS PER TABLE
SCALE: 8:1
SURFACE FINISH: UNFINISHED

TOLERANCES UNLESS OTHERWISE STATED:

X	±0.2
X.X	±0.1
X.XX	±0.05

REVISION HISTORY			
REV	DESCRIPTION	DATE	APPROVED
8.1	Dimensions Updated	13/11/2018	RY

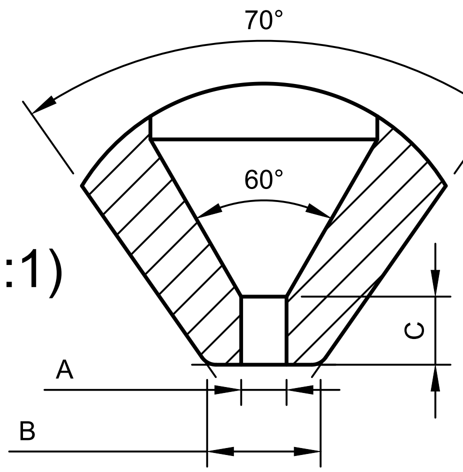

B (15:1)

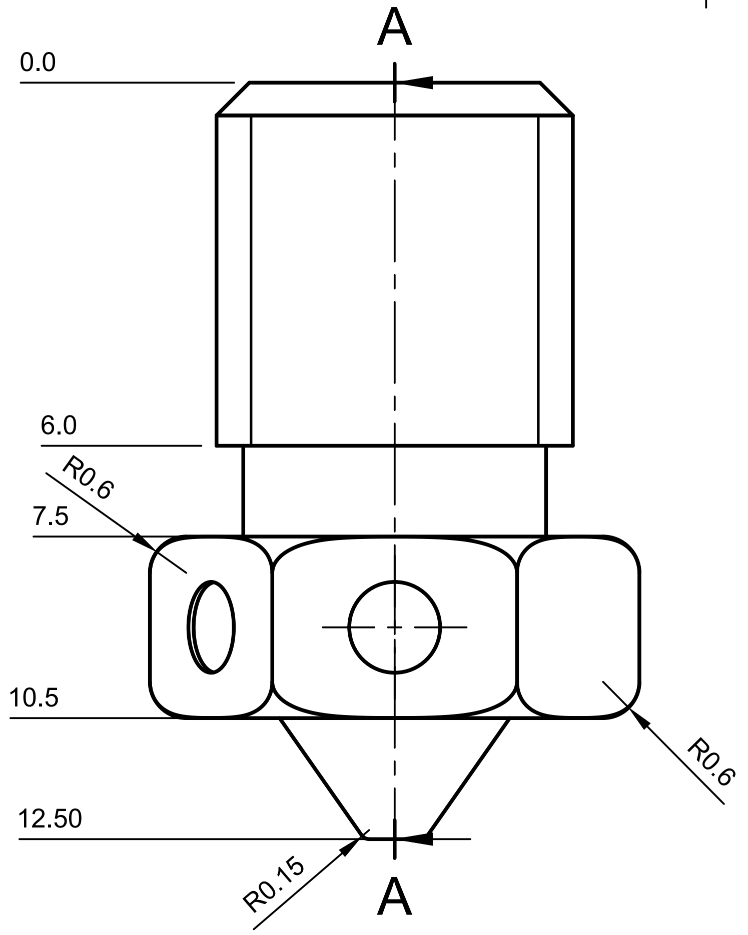

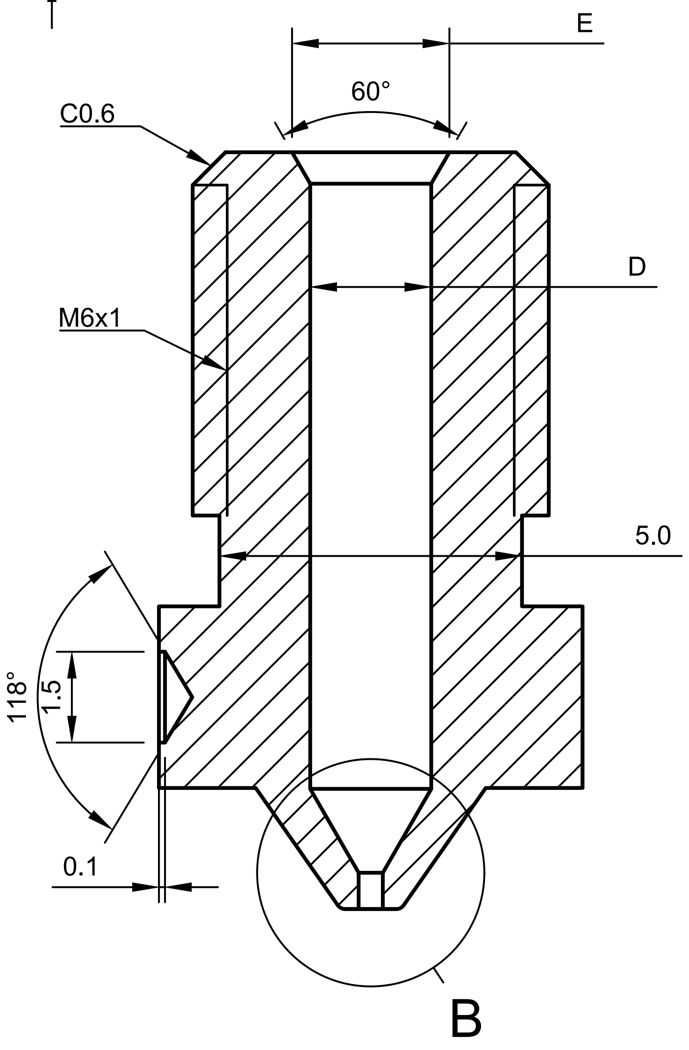

A-A (8:1)

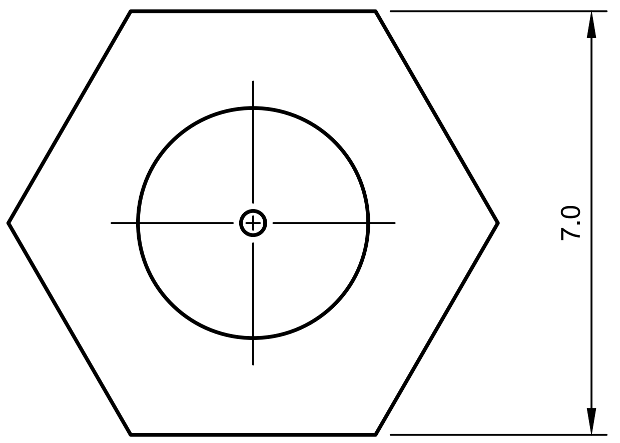

INLET GEOMETRY		
SKU CORE	D	E
-175-	2.0	2.6
-300-	3.2	3.9

TIP GEOMETRY				
SKU SUFFIX	A	B	C	# DOTS
150	0.15	0.30	0.18	2 on 1 FACE
250	0.25	0.63	0.38	0
300	0.30	0.75	0.45	1
350	0.35	0.88	0.53	2
400	0.40	1.00	0.60	3
500	0.50	1.25	0.90	6
600	0.60	1.50	1.20	4
800	0.80	2.00	1.60	5
000	UNDRILLED	1.00	0.60	0

Dept. Engineering	Technical reference V6	Created by RY 29/10/18	Approved by ST 29/10/18
		Document type PRODUCTION	Document status RELEASED
		Title V6 Nozzle Series	DWG No. V6-NOZZLE
			Rev. 8.1 · Date of issue 13/11/18 · Sheet 1/1
