# Supplementary material for: 3D Sugar Printing of Networks Mimicking the Vasculature
Source: Micromachines (Basel). 2019 Dec 30;11(1):43. doi: 10.3390/mi11010043 (PMC7019326; doi:10.3390/mi11010043)
Supplement: Supplementary file 1 [file micromachines-11-00043-s001.zip › Extruder assembly plate.pdf]

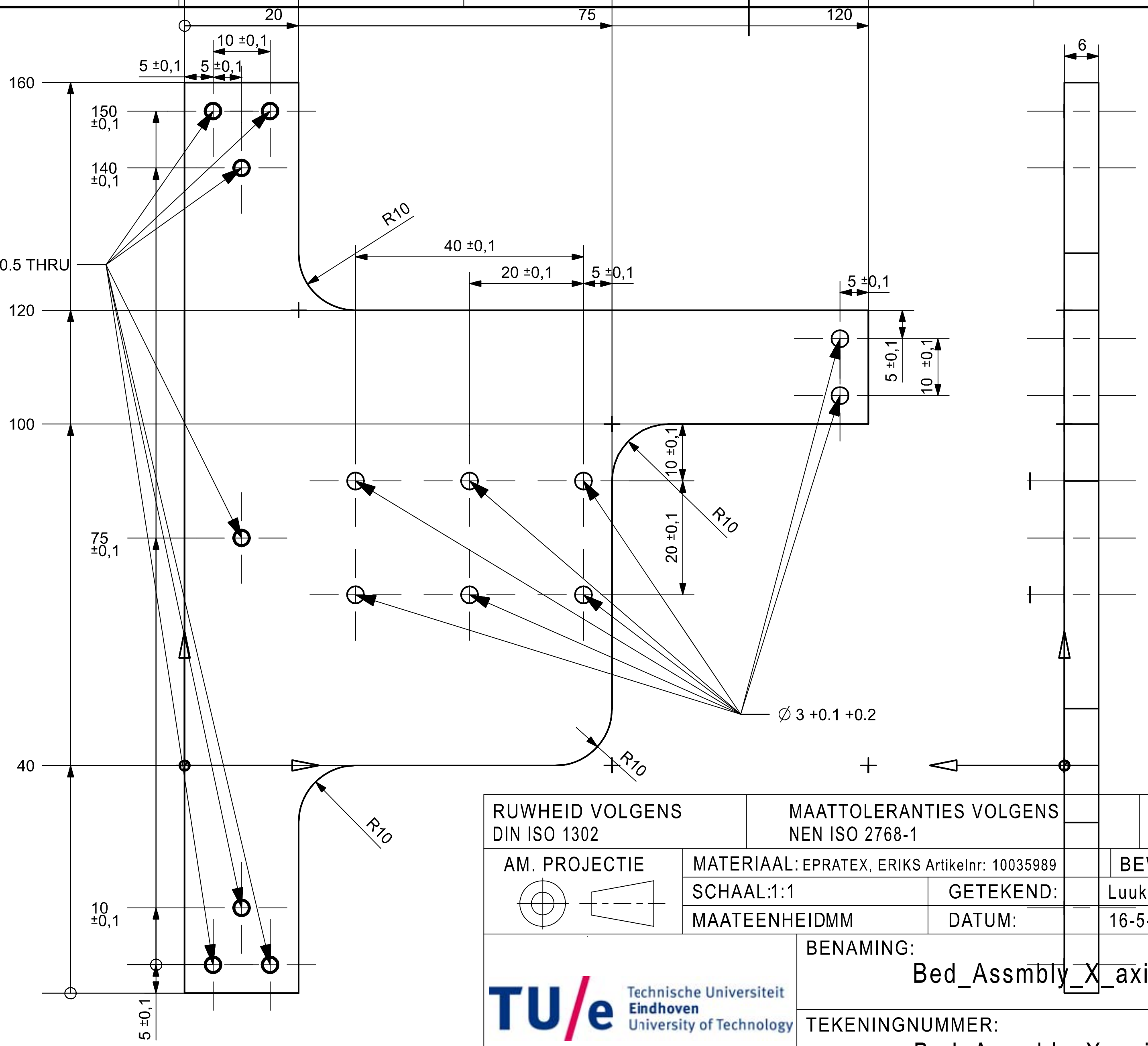

All dimensions without out a tolerance have a tolerance of +/- 0.5 mm

RUWHEID VOLGENS DIN ISO 1302	MAATTOLERANTIES VOLGENS NEN ISO 2768-1	VORM- EN PLAATSTOLERANTIES VOLGENS NEN ISO 1101

AM. PROJECTIE	MATERIAAL: EPRATEX, ERIKS Artikelnr: 10035989		BEWERKING:
	SCHAAL:1:1	GETEKEND:	Luuk Tooten — ID NUMMER: 0952315
	MAATEENHEIDMM	DATUM:	16-5-2018

Technische Universiteit Eindhoven University of Technology

BENAMING:
Bed_Assmbly_X_axis

TEKENINGNUMMER:	BLAD VAN BL.	FORMAAT:
Bed_Assmbly_X_axis-DRW	1 OF 1	A4
