# Supplementary material for: 3D Sugar Printing of Networks Mimicking the Vasculature
Source: Micromachines (Basel). 2019 Dec 30;11(1):43. doi: 10.3390/mi11010043 (PMC7019326; doi:10.3390/mi11010043)
Supplement: Supplementary file 1 [file micromachines-11-00043-s001.zip › Extruder barrel.pdf]

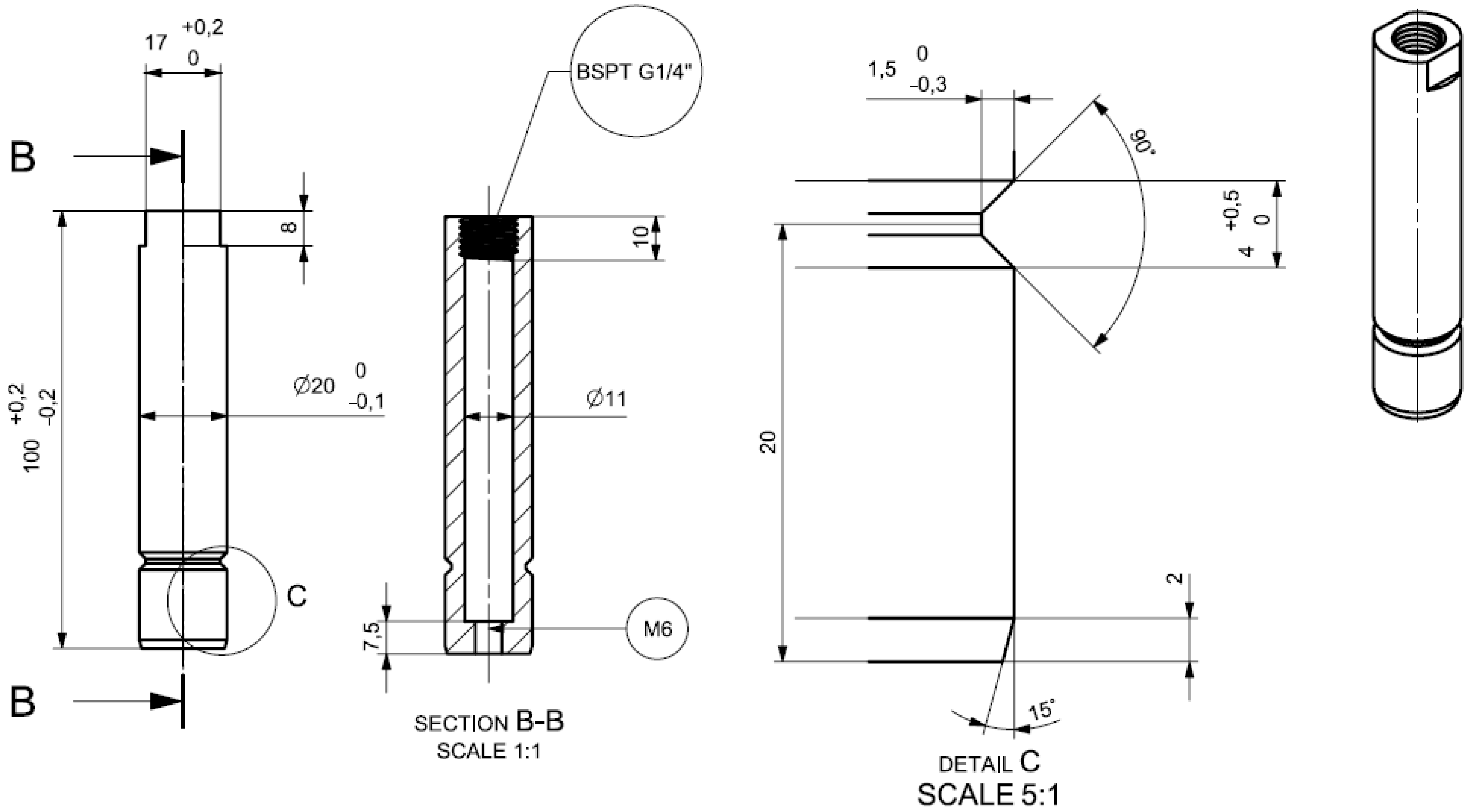

Remainder dimensions have tolerance of +/-0,5

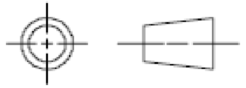

ALL DIMENSIONS IN MM

SIEMENS	THIS DRAWING HAS BEEN PRODUCED USING AN EXAMPLE TEMPLATE PROVIDED BY SIEMENS PLM SOFTWARE			
FIRST ISSUED	06-04-2018	TITLE		
DRAWN BY	Robert Dassen			
CHECKED BY				
APPROVED BY		SIZE A3	DRG NO. 9syringe	SHEET REV A
		SCALE 1:1		SHEET 1 OF 1

A3
