# Supplementary material for: 3D Sugar Printing of Networks Mimicking the Vasculature
Source: Micromachines (Basel). 2019 Dec 30;11(1):43. doi: 10.3390/mi11010043 (PMC7019326; doi:10.3390/mi11010043)
Supplement: Supplementary file 1 [file micromachines-11-00043-s001.zip › Extruder leveling mechanism.pdf]

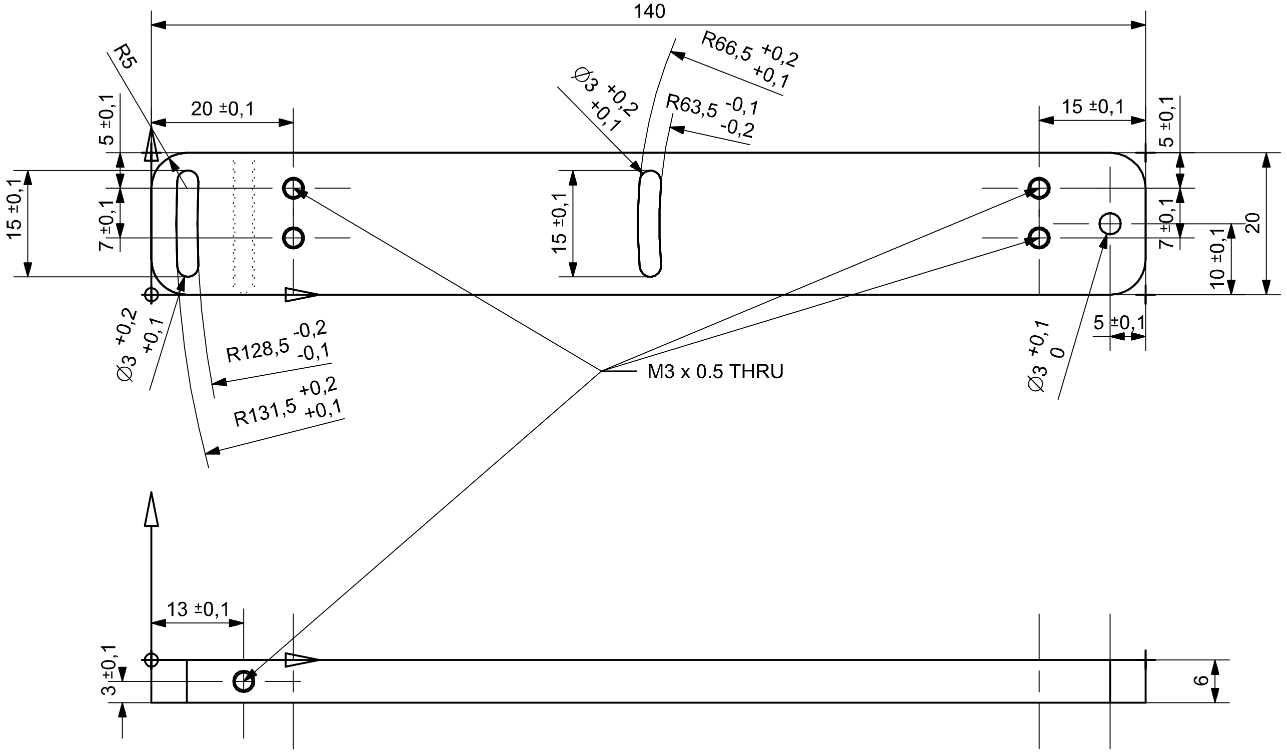

All dimensions without tolerances have a tolerance of +/- 0.5 mm

RUWHEID VOLGENS DIN ISO 1302	MAATTOLERANTIES VOLGENS NEN ISO 2768-1	VORM- EN PLAATSTOLERANTIES VOLGENS NEN ISO 1101	
AM. PROJECTIE	MATERIAAL: EPRATEX, ERIKS Artikelnr: 10035989	BEWERKING:	
	SCHAAL:1:1	GETEKEND: Luuk Tooten	ID NUMMER: 0952315
	MAATEENHEIDMM	DATUM: 16-5-2018	
TU/e Technische Universiteit Eindhoven University of Technology	BENAMING: Extruder_sideplate		
	TEKENINGNUMMER: Extruder_sideplate-DRW	BLAD VAN BL. 1 OF 1	FORMAAT: A4
