# Supplementary material for: 3D Sugar Printing of Networks Mimicking the Vasculature
Source: Micromachines (Basel). 2019 Dec 30;11(1):43. doi: 10.3390/mi11010043 (PMC7019326; doi:10.3390/mi11010043)
Supplement: Supplementary file 1 [file micromachines-11-00043-s001.zip › Materials list.docx]

2x Optische eindstop: <https://www.123-3d.nl/123-3D-Optische-eindstop-i90-t324.html>

2x GT2 Pulley hoge resolutie | 6 mm riem | 16 tanden | 5 mm as: <https://www.123-3d.nl/123-3D-GT2-Pulley-hoge-resolutie-6-mm-riem-16-tanden-5-mm-as-i2114-t14815.html>

2x GT2 Pulley hoge resolutie | met lager | 6 mm riem | 20 tanden | 5 mm as: <https://www.123-3d.nl/123-3D-GT2-Pulley-hoge-resolutie-met-lager-6-mm-riem-20-tanden-5-mm-as-i2084-t14815.html>

2x GT2 timing belt 6 mm (per meter): <https://www.123-3d.nl/123-3D-GT2-timing-belt-6-mm-per-meter-i40-t3046.html>

2x MGN12H lineaire slider blok: <https://www.123-3d.nl/123-3D-MGN12H-lineaire-slider-blok-i1620-t14800.html>

2x GT2 belt klem 40 mm: <https://www.123-3d.nl/123-3D-GT2-belt-klem-40-mm-i2417-t3046.html>

4x Riemspanner universeel: <https://www.123-3d.nl/123-3D-Riemspanner-universeel-i1554-t3046.html>

1x Voeding 12V (240V/115V / 30A max): <https://www.123-3d.nl/123-3D-Voeding-12V-240V-115V-30A-max-i104-t9746.html>

1x  Mean Well voeding 24V (150W, 6.5A) gesloten chassis: <https://www.123-3d.nl/Mean-Well-voeding-24V-150W-6-5A-gesloten-chassis-LRS-150F-24TE-i1702-t9747.html>

2x NEMA17 bevestigingsplaat / mount: <https://www.123-3d.nl/123-3D-NEMA17-bevestigingsplaat-mount-i1957-t14804.html>

2x NEMA17 motor beugel | bracket 90°: <https://www.123-3d.nl/123-3D-NEMA17-motor-beugel-bracket-90-i2129-t14804.html>

1x Megatronics v3.1 volledige geassembleerd: <https://www.123-3d.nl/123-3D-Megatronics-v3-1-volledig-geassembleerd-i2-t326.html>

2x NEMA17 HR stappenmotor | hoge resolutie 0,9 graden per stap | 4,0 kg/cm | 42BYGHM809: <https://www.123-3d.nl/123-3D-NEMA17-HR-stappenmotor-hoge-resolutie-0-9-graden-per-stap-4-0-kg-cm-42BYGHM809-i68-t14804.html>

2x Stepstick DRV8825 stappenmotordriver: <https://www.123-3d.nl/123-3D-Stepstick-DRV8825-stappenmotordriver-i96-t332.html>

5x Keramische verwarmingselementen 12v/40w: <https://www.123-3d.nl/123-3D-Keramisch-verwarmingselement-heater-cartridge-12V-40W-150-cm-i2274-t394.html>

2x Thermokoppel K-type: <https://www.123-3d.nl/123-3D-Thermokoppel-type-K-gelaste-tip-glas-fiber-i112-t3117.html>

1x Ventilator <https://www.123-3d.nl/123-3D-Ventilator-12V-50x15mm-radiaal-i1709-t15050.html>
