# Supplementary material for: 3D Sugar Printing of Networks Mimicking the Vasculature
Source: Micromachines (Basel). 2019 Dec 30;11(1):43. doi: 10.3390/mi11010043 (PMC7019326; doi:10.3390/mi11010043)
Supplement: Supplementary file 1 [file micromachines-11-00043-s001.zip › Printbed assembly plate bottom.pdf]

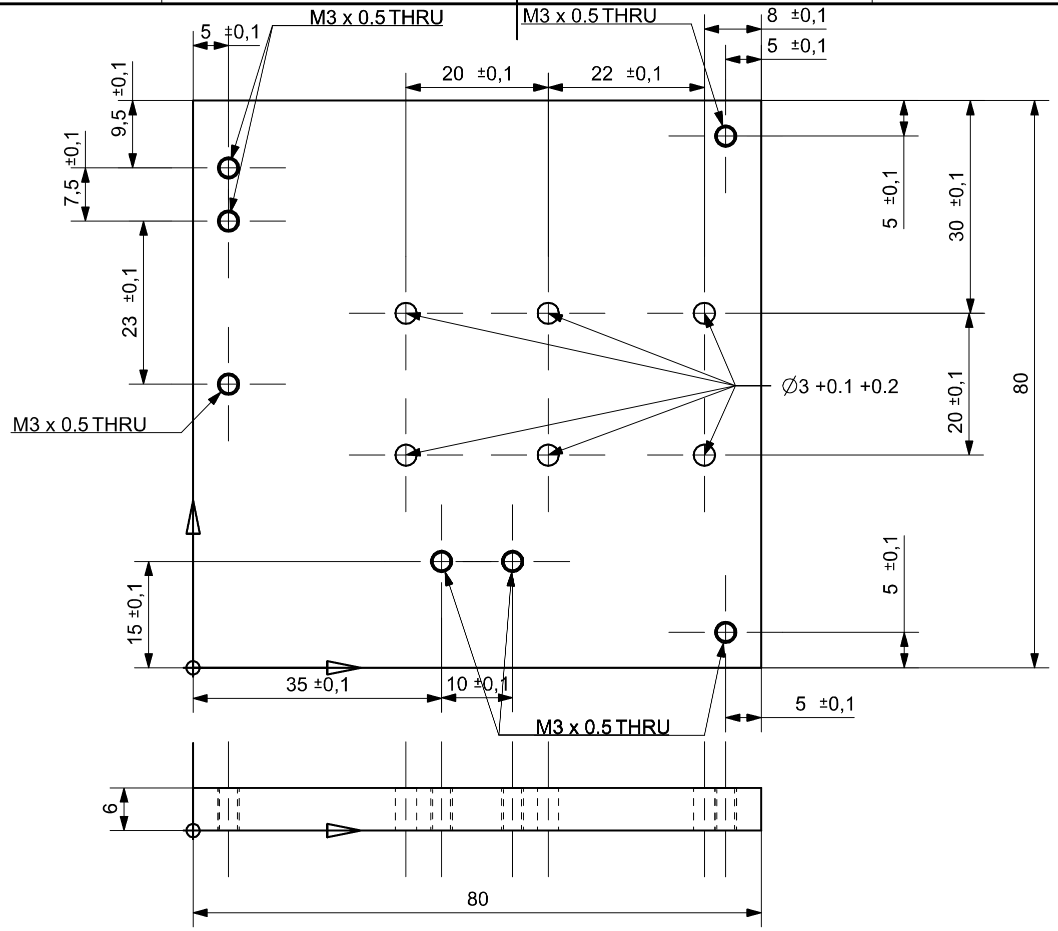

All dimensions without a tolerance have a tolerance of +/- 0.5 mm

RUWHEID VOLGENS DIN ISO 1302	MAATTOLERANTIES VOLGENS NEN ISO 2768-1	VORM- EN PLAATSTOLERANTIES VOLGENS NEN ISO 1101

AM. PROJECTIE

MATERIAAL: EPRATEX, ERIKS Artikelnr: 10035989

BEWERKING:

SCHAAL: 1:1

GETEKEND: Luuk Tooten

ID NUMMER: 0952315

MAATEENHEID MM

DATUM: 16-5-2018

Technische Universiteit Eindhoven University of Technology

BENAMING: Bed_Assmbly

TEKENINGNUMMER: Bed_Assmbly-DRW

BLAD VAN BL. 1 OF 1

FORMAAT: A4
